# Novel Aggregation-Induced Emission Materials/Cadmium Sulfide Composite Photocatalyst for Efficient Hydrogen Evolution in Absence of Sacrificial Reagent

**DOI:** 10.3390/ma13225287

**Published:** 2020-11-22

**Authors:** Xi Ke, Kunqiang Wang, Chen Tu, Runda Huang, Dongxiang Luo, Menglong Zhang

**Affiliations:** 1 Institute of Semiconductors, South China Normal University, Guangzhou 510631, China; xike@m.scnu.edu.cn (X.K.); kunqiangwang@m.scnu.edu.cn (K.W.); 2 School of Chemistry, Faculty of Science, Chemistry Building F11, Camperdown 2050, University of Sydney, Camperdown, NSW 2006, Australia; chtu4116@uni.sydney.edu.au; 3 School of Materials and Energy, Guangdong University of Technology, Guangzhou 510006, China; hrd76287211@163.com

**Keywords:** aggregation-induced emission luminogens, photocatalysis, photoelectrochemistry

## Abstract

This work focuses on the development of a novel organic–inorganic photoactive material composited by aggregation-induced emission luminogens (AIE) and CdS. Tetraphenylethene-based AIE (TPE-Ca) is synthesized on CdS to form CdS/TPE-Ca electrode, due to its suitable band structure and potential capability of renewable energy production. The CdS/TPE-Ca electrode presents over three-fold improved photocurrent density and dramatically reduced interfacial resistance, compared with the pure CdS electrode. In addition, the engineering of the band alignment allows the holes to accumulate on the valance band of TPE-Ca, which would partially prevent the CdS from photo-corrosion, thus improving the stability of the sacrificial-free electrolyte photoelectrochemical cell.

## 1. Introduction

As a valuable subgroup of semiconductive photocatalysts, transition-metal sulfides typically possess narrower band gaps than that of metal oxides, which makes them capable of various useful redox reactions under mild conditions, for instance, water splitting [1,2], high-energy-density supercapacitors [3,4], lithium-sulfur batteries [5], sodium ion battery anodes [6], for photovoltaic and photoelectrochemical devices [7]. To further explore the utilizations of the relevant photocatalysts, metal sulfides have been cooperated with many kinds of materials including metal oxides [8], carbon materials [9,10], and metal particles [11].

Among transition-metal sulfides, CdS and ZnS have very frequently been exploited as photocatalysts for heterogeneous photocatalytic reactions for several decades [12]. In detail, CdS is a direct bandgap semiconductor with suitable band position and bandgap energy of ca. 2.4 eV, corresponding to an absorption range up to 520 nm. In addition, CdS is cheap and commonly easy for synthesis. These features make it one of the most attractive semiconductive photocatalysts active to visible light. Most attention to CdS has been focused on solar energy conversion [13,14,15], selective organic transformation [16,17] and degradation of environmental pollutants [18,19,20]. However, one critical issue, photo-corrosion, has severely hampered its developments in addressing the pending environmental and energy challenges. For instance, in the absence of an S^2–^ aqueous sacrificial reagent in the photoelectrochemistry cell (PEC), the orange CdS photoelectrode would turn to pale yellow within several minutes. The photogenerated holes oxidize S^2–^ in CdS rather than H_2_O, accompanied with the evolution of Cd^2+^ and S [21]. Typically, a sacrificial reagent containing S^2–^ is commonly exploited to prevent the photo-corrosion of metal sulfides during the photocatalytic redox reactions [22,23,24,25,26,27], in which the S^2–^ is consumed as a sacrificial reagent instead of CdS. An alternative strategy to hamper the photo-corrosion of CdS is constructing suitable heterostructure photocatalysts, to transfer the photo-generated holes to the valance band (VB) of the coupled materials. This approach will not only protect the CdS, but can also potentially enhance the charge separation and light absorption. As previously reported, CdS-involved heterostructure photocatalysts have presented promising stability and photoactivity performances. Very recently, composite materials of metal sulfides coupled with metal-organic frameworks (MOFs) [28,29,30], perovskites [31,32], and graphitic carbon nitride (g-C_3_N_4_) [33,34] were developed for applications in hydrogen evolution reaction (HER), oxygen evolution reaction (OER) and photovoltaic devices. However, examples of heterostructure coupled with CdS and organic materials are still rare. In addition, it has been demonstrated that the metal sulfides are customarily in direct contact with organic substrates.

Basically, the photocatalytic mechanism involves three steps: (1) the photoactive materials capture the photon with greater energy than their band gap energies, (2) subsequently, they generate photoexcited electrons and holes in the conduction and valence bands, respectively, (3) the photogenerated electrons and holes would further be transported to the reaction site for redox reactions. However, it is unavoidable that part of the photogenerated electrons and holes would recombine with each other and emit photons during these processes, thus reducing the photocatalytic activity. The approaches to reduce the recombination for inorganic photocatalysts can be summarized as optimizing the defects of materials, reducing the pathway necessary for charge carrier migration, and fabricating heterostructure materials to improve the separation of charge carrier [35]. To improve the photocatalytic efficiency of organic photocatalysts, the key approach is to reduce the non-radiation pathway and transfer the photogenerated electrons and holes. As we know, the heterogeneous structures, such as alkalinized C_3_N_4_, TiO_2_-based hybrid materials, would play a significant role in consuming photogenerated electron-holes through a non-radiation process [36,37].

Tang’ group developed a series of novel luminogens, defined as ‘aggregation-induced emission’ (AIE) in 2001. The AIE materials typically exhibited an abnormal photophysical phenomenon. In detail, the AIE materials presents dramatically enhanced fluorescence intensity of outstanding quantum yield up to 100% in aggregate state (in solid state). In terms of the work mechanism of AIE, for instance, in hexaphenylsilole (HPS), the peripheral phenyl rings tend to freely rotate around the C-C single bond relative to the central silacyclopentadiene or silole plane, resulting in a non-radiation process with weak emission, while in the aggregation state, partial suppression of molecular rotation would occur, attributed to the intramolecular interactions. Then, the non-radiative decay channels would be inhibited by the restriction of intramolecular rotations (RIR). Therefore, the radiative decay pathway would be available, leading to the observed AIE phenomena. For the solid-state AIE materials, although they possess a promising light-capturing capability, the strong recombination should be suppressed before being exploited as an excellent candidate for photocatalysis reactions. Previously, Feng et al. demonstrated that a tailored cyanostilbene-based molecule (TPE-Ca) with AIE feature, which showed promising photocatalytic activity in organic pollutants’ degradation [20]. The suitable band structure and good stability of TPE-Ca inspired us to construct a heterostructure photocatalyst with CdS, extending its applications in the solar energy field.

In this work, we report a novel CdS/TPE-Ca heterostructure photocatalyst with outstanding photoactivity and stability for hydrogen evolution in the absence of a sacrificial reagent. By utilizing the relative band alignment of charge carriers in CdS/TPE-Ca composites, the optimized photocatalytic performance was acquired. The photoelectrochemical features of the composite photocatalyst is carried out and found to benefit from the rapid charge carrier’s migration.

## 2. Experimental

### 2.1. Chemicals

#### 2.1.1. Synthesis of CdS on Fluorine Doped tin Oxide (FTO) Glass

FTO glass (10 × 15 mm) was immersed in a solution of Cd(Ac)_2_ (50 mM, dissolved in ethanol) for 1 min. The FTO glass was then removed from the solution and dried under a stream of N_2_. Subsequently, the FTO glass was immersed in Na_2_S solution (50 mM, aqueous) for another 1 min. The FTO glass was then rinsed by distilled water and dried by a stream of N_2_. This reaction cycle was repeated five times. Finally, the FTO glass was transferred into a column oven, and heated at 400 °C for 30 min using a ramp rate of 1 °C min^−1^ under argon atmosphere. The as-prepared CdS electrode exhibited an orange color.

#### 2.1.2. Synthesis of AIE Materials (TPE-Ca)

**Scheme 1:**
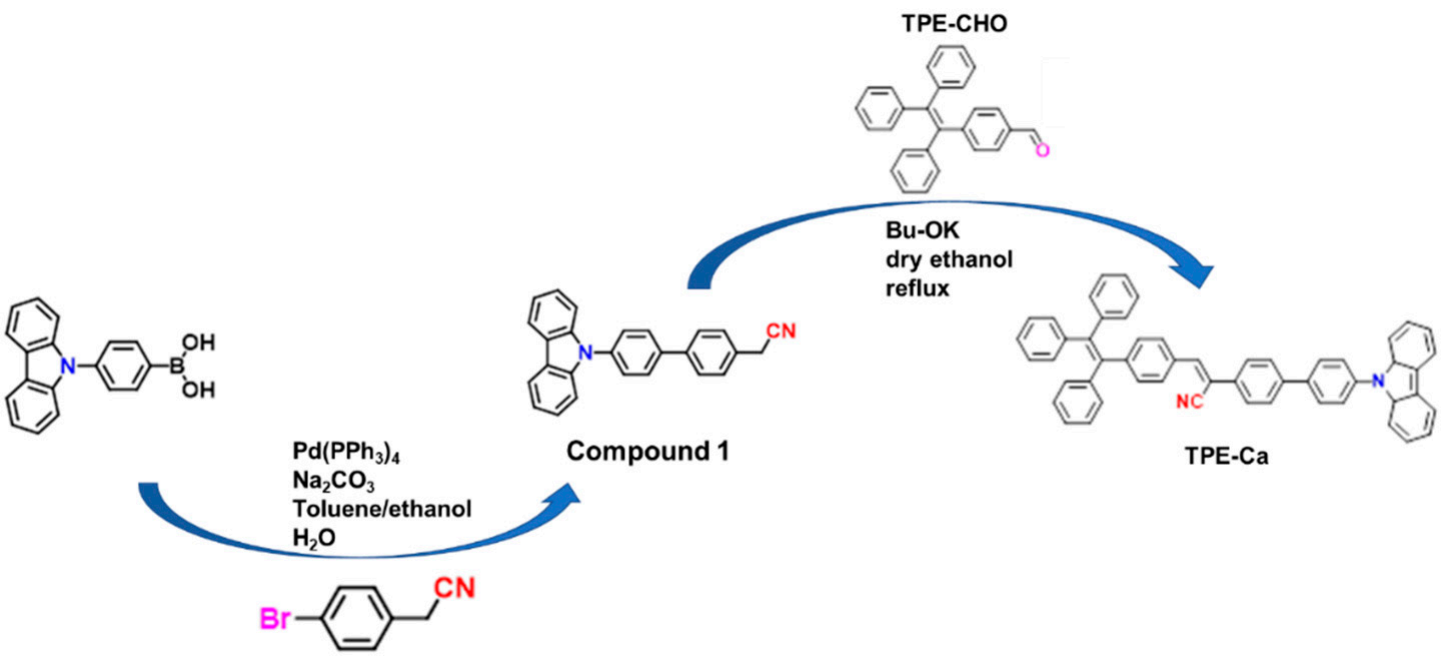
The process for the synthesis of TEP-Ca.

The synthesis of TPE-Ca was referred to in one of our previous reports [38]. In detail, 2-(4′-(8a,9a-dihydro-9H-carbazol-9-yl)-[1,1′-biphenyl]-4-yl) acetonitrile (**Compound 1** in Scheme 1) was initially synthesized as the AIE precursor. A mixture of 2-(4-bromophenyl)acetonitrile (400 mg, 2.04 mmol, 1.00 equiv), 9H-Carbazole-9-(4-phenyl) boronic acid (700 mg, 2.44 mmol, 1.20 equiv), sodium carbonate (500 mg, 4.71 mmol, 2.3 equiv) and tetrakis(triphenylphosphine) palladium (40 mg, 0.035 mmol, 0.02 equiv) in toluene/ethanol (10 mL/3 mL) was stirred and refluxed overnight under nitrogen atmosphere. The mixture was then stirred and maintained at 90 °C for 24 h before naturally cooling to ambient temperature. Subsequently, the mixture was quenched by DI water, and then the CH_2_Cl_2_ (100 mL three times) was employed for extraction. After washing with water and brine, the organic extracts were dried by MgSO_4_ and evaporation in vacuum oven. The purification of the residue was achieved using column chromatography with CH_2_Cl_2_/hexane mixture (V_CH2Cl2_:V_hexane_ = 1:1) as an eluent to obtain **Compound 1** (light white powder, 450 mg, yield 61%).

**Compound 1** (240 mg, 2.09 mmol, 1.20 equiv), 4-(1,2,2-triphenylvinyl)benzaldehyde (TPE-CHO) (627 mg, 1.74 mmol, 1.00 equiv) and potassium t-butoxide (380 mg, 2.09 mmol, 1.20 equiv) were put into a round-bottom flask (100 mL) under N_2_ asmosphere. The mixture was refluxed overnight after adding with 30 mL ethanol. After cooling to ambient temperature, the mixture was filtered and washed with ethanol three times. At last, the residue was transferred in CH_2_Cl_2_ and hexane for crytallization, giving the final product (yellow powder, 327 mg, yield 70%). The detailed reaction process was illustrated below in Scheme 1.

#### 2.1.3. Preparation of CdS/TPE-Ca Electrode

A total of 5 mg of TPE-Ca was added into 10 mL hexane/CH_2_Cl_2_ mixture (1:1) solvent with stirring for 10 min (0.5 mg/mL) in a glass vial (10 mL). Subsequently, the as-prepared CdS electrode was stood vertically in the TEP-Ca solution under 60 °C for 4 h to obtain the CdS/TPE-Ca electrode.

### 2.2. Characterization

A JEOL 2011 transmission electron microscope (Tokyo, Japan) operated at 200 kV accelerating voltage was employed for the TEM measurements. Samples for TEM tests were ground and ultra-sonicated in ethanol. A drop of the dispersion was put onto an ultra-thin carbon, copper grid. Diffuse-reflectance spectra were recorded using an Ocean Optics HR2000+ High Resolution Spectrometer (Largo, FL, USA), with a light source (200–1100 nm) supplied by DH-2000-BAL Deuterium/Helium. Spectra were recorded in Spectra Suite software, using the following conditions: 10 s integration time, 30 width box car smoothing and 10 scans on average. PXRD patterns were measured using a Bruker-AXS D8 Advance (Karlsruhe, Germany) instrument with Lynx eye detector, with Cu Kα radiation, scanning from 10 to 80° (2θ), with a 0.02° step size. The data were recorded for every 0.05 s.

Photoelectrochemical measurements were carried out in a standard three-electrode cell in which Ag/AgCl (3 M NaCl internal solution) was utilized as the reference electrode, and a platinum wire was used as the counter electrode. The working electrode (sample) was connected by copper tape. A working area of 1 cm^2^ was obtained by immersing the bottom 1 cm of the working electrode in the electrolyte solution. The photoelectrochemical cell was back-illuminated through a Pyrex window with a long-pass filter (>400 nm). The simulated solar light was obtained using a 150 W Xe lamp with an irradiance of 100 mW cm^−2^. Electrolyte solutions were prepared using water filtered using a Millipore system (>18 MΩ cm^−3^), and degassed for 10 min with N_2_ before use. Electrodes coated with CdS or CdS/TPE-Ca were measured in an electrolyte of aqueous Na_2_S_(aq)_/Na_2_SO_3(aq)_ (0.25/0.35 M) (pH 13) or NaOH/Na_2_SO_4(aq)_ (pH 13).

## 3. Results and Discussions

### 3.1. Crystal Structure of CdS/TPE-Ca

**Figure 1:**
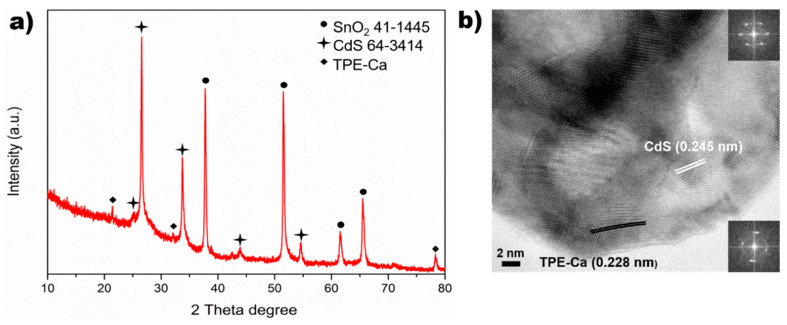
(**a**) The PXRD patterns of the as-prepared CdS/TPE-Ca electrode; (**b**) the HR-TEM and SAED of the CdS/TPE-Ca scrapped from one electrode.

Powder X-ray diffraction (PXRD) was initially carried out to check the as-prepared CdS/TPE-Ca electrode. As shown in Figure 1a, the diffraction patterns can be corresponded to the FTO substrate (SnO_2_, JCPDS 41-1445) and CdS (JCPDS 64-3414). It should be noted that FTO typically presented slightly shifted diffraction patterns due to the doped fluorine ions [39]. The crystal structure of TPE-Ca (CCDC 1914539) [38] was analysed by X-ray single-crystal diffraction, which revealed a monoclinic crystal structure with space group P1 21/n 1. The detailed parameters of TPE-Ca can be found in Table S1 (Supporting Information). Typically, the non-planar conformation would lead to a close-packed matrix, thus inhibiting intramolecular rotation and enhanced light harvesting capability. In addition, high-resolution transmission electron microscopy (HR-TEM) was exploited to identify the CdS/TPE-Ca, in which the lattice fringes and selected area electron diffraction (SAED) results were consistent with the crystal structures of CdS and TPE-Ca, respectively.

### 3.2. Optical Features

**Figure 2:**
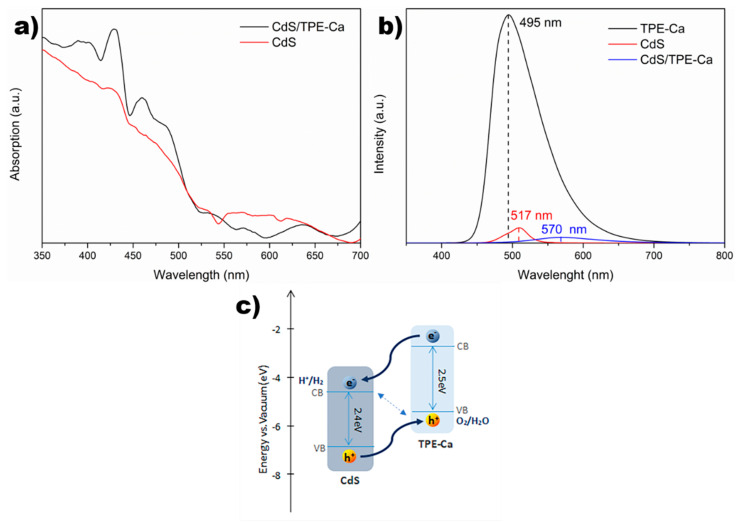
(**a**) DRUVS spectra of CdS and CdS/TPE-Ca photocatalysts, the absorption was approximated by the Kubelka-Munk function via diffuse reflectance, the unit of the two samples are the same; (**b**) PL spectra of TPEC-Ca, CdS and CdS/TPE-Ca photocatalysts. For the PL measurement, the samples were irradiated by a 50 mW 405 nm steady-laser with 1% output power. (**c**) Proposed band alignment of CdS/TPE-Ca.

The optical features of CdS/TPE-Ca electrode was demonstrated using diffuse reflectance UV-visible spectra (DRUVS) and photoluminescence (PL) spectra. As illustrated in Figure 2a, the absorption edge CdS was located at ca. 500 nm, suggesting its promising capacity for light absorption in the visible range. Next, after compositing with TPE-Ca, the CdS/TPE-Ca presented stronger absorbance in the range shorter than 450 nm, in comparison to that of pure CdS. The absorption in this range can be further enhanced by increasing the concentration of TPE-Ca solution during the preparation. Two absorption peaks appeared at ca. 425 and 460 nm, possibly due to the light absorption of peripheral phenyl rings, leading to intramolecular rotations; this phenomenon was also observed in other AIEs [40]. In addition, as shown in Figure 2b, TPE-Ca exhibited a very intensive PL maximum centred at 495 nm, corresponding to a band gap energy of ca. 2.5 eV. It should be noted that the measured band gap energy is smaller than that obtained by the density functional theory (DFT) calculation (ca. 3.0 eV). On the other hand, the CdS presented its PL maximum at 517 nm, corresponding to a band gap energy of ca. 2.4 eV. After incorporating CdS with TPE-Ca, the composite photocatalyst revealed a dramatically reduced PL intensity located at 570 nm. This result suggested that, although the overall recombination is reduced, there is still a partial recombination of the electrons from the conduction band (CB) of CdS and the holes from the valance band (VB) of TPE-Ca.

### 3.3. Photoelectrochemical Cell

**Figure 3:**
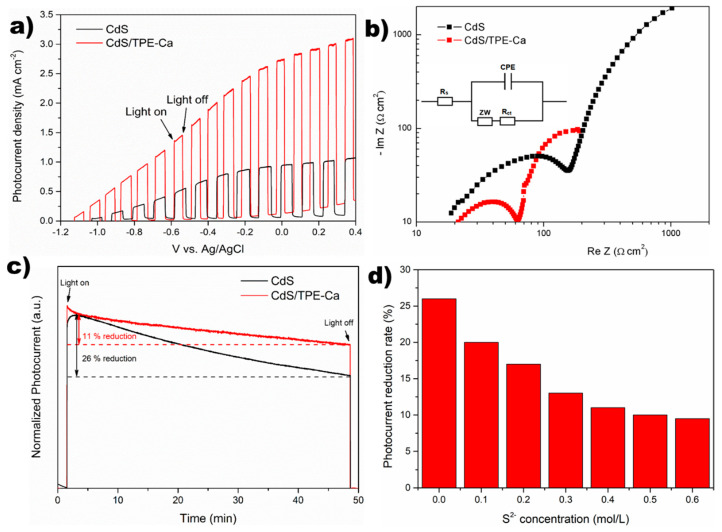
(**a**) Linear sweep voltammogram with chopped illumination every 10 s and (**b**) electrochemical impedance spectroscopy (EIS) Nyquist plots of CdS and CdS/TPE-Ca electrodes under illumination, respectively; (**c**) the photostability test of CdS and CdS/TPE-Ca in NaOH/Na_2_SO_3_ (pH 13) electrolyte, the photocurrent density of photoelectrodes were normalized for convenient comparison; (**d**) the photocurrent reduction rate of CdS after 50 min illumination when using different concentration of Na_2_S sacrificial reagent.

Linear sweep voltammetry (LSV) measurements were carried to evaluate the photoactivity of the as-prepared electrodes. The simulated solar source is obtained using Xe lamp (100 mW cm^−2^), which was chopped for every 10 s. The samples were employed as the working electrode in the standard three-electrode photoelectrochemical cell. The CdS/TPE-Ca displayed remarkably improved photoactivity comparedwith the pure CdS electrode. In detail, the CdS/TPE-Ca electrode had a photocurrent density of ca. 2.55 mA cm^−2^ at bias of 0 V vs. Ag/AgCl, which can be compared to that from the pure CdS electrode of ca. 0.72 mA cm^−2^ under the same conditions. This could be attributed to the increased light absorption and the reduced recombination of the composited photocatalyst. In addition, the CdS/TPE-Ca revealed lower on-set potential (ca. −1.1 V vs. Ag/AgCl) compared with pure CdS electrode (ca. −1.0 V vs. Ag/AgCl), due to the higher conduction band of TPE-Ca. The photocurrent density of CdS/TPE-Ca (2.55 mA cm^−2^) can be compared to the recent developed CdS/MoS_2_ (1.9 mA cm^−2^) [41], CdS/WO_x_ (10 μA cm^−2^) [42], and CdS nanowires (2.0 mA cm^−2^) [43]. Subsequently, the electrochemical impedance spectroscopy (EIS) Nyquist plots was conducted to demonstrate the electrochemical kinetics toward the interfaces between samples and electrolyte. The CdS/TPE-Ca presented much smaller arc radius on EIS Nyquist plot, suggesting that the interfacial resistance and the limits of electron transfer were dramatically reduced by coupling the TEP-Ca with CdS. In detail, as illustrated in the fitted equivalent circuit (Figure 3b), the series resistance (R_s_) was measured to be ca. 20 Ω for CdS and CdS/TPE-Ca. On the other hand, in the semicircle portion, the interfacial electron transfer resistance (R_ct_) was measured to be ca. 167 Ω and 34 Ω for CdS and CdS/TPE-Ca, respectively. It should be noted that, in the lower frequency region, the linear portion (commonly exhibits as an arc due to Dispersion effect [44]) represented a diffusion-limited electron transfer process (Z_w_) [45]. Next, the photostability of the CdS/TPE-Ca electrode is measured under illumination for 50 min in the absence of S^2–^ (Figure 3c); only ca. 11% reduction of photocurrent density is observed, and the photo-corrosion rate is much slower than our pristine CdS electrode (reduced ca. 26% in 50 min) and CdS/WO_x_ composite (reduced ca. 35% in 50 min) [24] photocatalyst in the absence of S^2–^. These results suggested that the TPE-Ca can efficiently collect the holes from the valance band of CdS and thus hamper the photo-corrosion. At last, the photocurrent reduction rate in S^2–^ sacrificial reagent of different concentrations was tested (Figure 3d). An obvious improvement was observed when adding 0.1 M sacrificial reagent. In addition, the CdS photoelectrode in 0.3 M Na_2_S exhibited a similar photocurrent reduction rate, compared with the CdS/TPE-Ca photoelectrode. On the other hand, the photostability could not be further improved by increasing the concentration over 0.5 M. This result suggested that although the photo-corrosion was a key factor for the photostability of transition-metal sulfides, other factors should also be responsible. For instance, the gas bubbles attached on the electrodes would decrease the active surface area, and the increased temperature of photoelectrode when under long time illumination would also affect the photocatalytic performance.

## 4. Conclusions

In summary, a novel organic-inorganic composite photocatalyst (CdS/TPE-Ca) was successfully fabricated. The as-prepared CdS/TPE-Ca electrode exhibited several improved photocatalytic performances compared with the pure CdS electrode: two-fold enhanced photocurrent density was obtained, as well as 0.1 V lower on-set potential necessary for photocurrent generation, optimized interfacial conductivity and, more importantly, the sample showed good stability in the absence of an S^2–^ sacrificial reagent; the photocurrent loss is only 11% in the absence of a sacrificial reagent. Apart from the nature of the two materials, the suitable band alignment and the optimized interfacial resistance should be responsible for these features. We believe this work could give some insights for utilizations of AIE materials in the field of photocatalysis.

## Supplementary Materials

The following are available online at https://www.mdpi.com/1996-1944/13/22/5287/s1, Figure S1: Frontier molecular orbital distributions and energy level diagram of TPE-Ca obtained by DFT calculations (B3LYP/6–31G*), Table S1: The main parameters of TEP-Ca.
