# Supplementary material for: Novel Aggregation-Induced Emission Materials/Cadmium Sulfide Composite Photocatalyst for Efficient Hydrogen Evolution in Absence of Sacrificial Reagent
Source: Materials (Basel). 2020 Nov 22;13(22):5287. doi: 10.3390/ma13225287 (PMC7700582; doi:10.3390/ma13225287)
Supplement: Supplementary file 1 [file materials-13-05287-s001.pdf]

materials

MDPI

Supplementary Materials

Novel Aggregation-Induced Emission Materials/Cadmium Sulfide Composite Photocatalyst for Efficient Hydrogen Evolution in Absence of Sacrificial Reagent

Xi Ke [1], Kunqiang Wang [1], Chen Tu [2], Runda Huang [3], Dongxiang Luo [1,*] and Menglong Zhang [1,*]

[1] Institute of Semiconductors, South China Normal University, Guangzhou 510631, China; xike@m.scnu.edu.cn (X.K.); kunqiangwang@m.scnu.edu.cn (K.W.)
[2] School of Chemistry, Faculty of Science, Chemistry Building F11, Camperdown 2050, University of Sydney, Camperdown NSW 2006, Australia; chtu4116@uni.sydney.edu.au
[3] School of Materials and Energy, Guangdong University of Technology, Guangzhou 510006, China; hrd76287211@163.com
* Correspondence: luodx@gdut.edu.cn (D.L.); mlzhang@m.scnu.edu.cn (M.Z.)

DFT Calculation of Band Structure of TEP-Ca

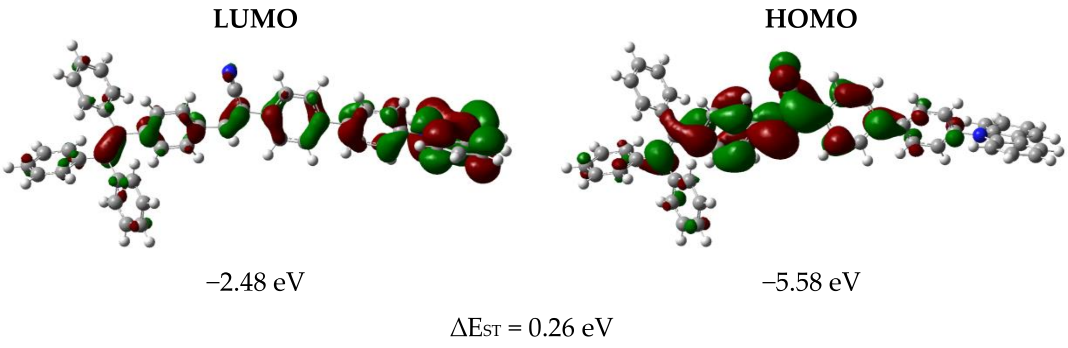

Figure S1. Frontier molecular orbital distributions and energy level diagram of TPE-Ca obtained by DFT calculations (B3LYP/6–31G*).

X-Ray Single-Crystal Diffraction of TEP-Ca

Table S1. The main parameters of TEP-Ca.

Parameter	TPE-Ca
Empirical formula	$C_{53}H_{36}N_2$
Formula weight[g mol^{-1}]	700.84
Crystal system	monoclinic
Space group	P 1 21/n 1
A [Å]	19.8975(12)
B [Å]	9.0425(6)
C [Å]	21.1651(13)
α [°]	-
β[°]	92.499(2)
γ[°]	--
Volume [Å^3]	3804.5(4)
Z	4
Density, calcd [g m^{-3}]	1.224
Temperature [K]	220
Unique reflns	6845
Obsdreflns	7994
Parameters	496
R_{int}	0.0636
R[I > 26(I)][a]	0.1698
wR[I > 2(I)]l[b]	0.1830
GOF on F^2	1.024

© 2020 by the authors. Submitted for possible open access publication under the terms and conditions of the Creative Commons Attribution (CC BY) license (http://creativecommons.org/licenses/by/4.0/).
